# Quality of life impairment in patients with head and neck cancer and their caregivers: a comparative study^☆^

**DOI:** 10.1016/j.bjorl.2015.12.012

**Published:** 2016-04-11

**Authors:** Laís Rigoni, Raphaella Falco Bruhn, Rafael De Cicco, Jossi Ledo Kanda, Leandro Luongo Matos

**Affiliations:** a Faculdade de Medicina do ABC, Santo André, SP, Brazil; b Faculdade de Medicina do ABC, Disciplina de Cirurgia de Cabeça e Pescoço, Santo André, SP, Brazil; c Faculdade de Medicina do ABC, Disciplina de Saúde Coletiva (Curso de Bioestatística), Santo André, SP, Brazil

**Keywords:** Squamous cell carcinoma, Quality of life, Caregivers, Head and neck cancer, Carcinoma espinocelular, Qualidade de vida, Cuidadores, Câncer de cabeça e pescoço

## Abstract

**Introduction:**

Head and neck cancer represents 3% of all the types of malignant neoplasms and squamous cell carcinoma (SCC) is responsible for 90% of these cases. There have been some studies evaluating the quality of life of these patients, but little is known about the physical and emotional effects on their caregivers.

**Objective:**

To evaluate the quality of life of patients with head and neck cancer and their caregivers by applying validated questionnaires.

**Methods:**

Thirty patients with advanced tumors (SCC stage III or IV) and their 30 caregivers were included. Specific questionnaires (Coop/Wonca, EORTC QLQ–C30, EORTC H&N35, Coop/Wonca, and Caregiver Strain Index – CSI) were applied during routine medical consultations.

**Results:**

Of the 30 patients, 28 were males and 25 had stage IV tumors, with mean age of 56.6 years. 36.7% had the primary tumor in the oropharynx and 70% reported pain. The functional cognitive, physical, and emotional scales were the most affected. Pain, fatigue, and sleep disorders were the most prevalent symptoms. Of the 30 caregivers, 23 were females and 70% were the primary caregivers. 36.7% of the caregivers had high levels of stress, mainly related to the feeling of incapacity. The comparison between patients and caregivers demonstrated that the two groups had similar quality of life impairment: physical fitness (*p* = 0.487), mental health (*p* = 0.615), daily activities (*p* = 0.793), social activities (*p* = 0.301), changes in health (*p* = 0.649), and overall health (*p* = 0.168).

**Conclusion:**

Quality of life impairment is similar between patients and their caregivers. This result demonstrates that not only the patients show quality of life impairment, but their caregivers also have it and at similar proportions.

## Resumo

**Introdução:**

O câncer de cabeça e pescoço representa 3% de todos os tipos de neoplasias malignas e o carcinoma espinocelular corresponde a 90% dos casos. Há estudos sobre a qualidade de vida desses pacientes, mas pouco se conhece sobre os prejuízos físico e emocional dos seus cuidadores.

**Objetivo:**

Avaliar a qualidade de vida de pacientes com câncer de cabeça e pescoço e comparativamente de seus cuidadores a partir da aplicação de questionários validados.

**Método:**

A casuística foi constituída de 30 pacientes com tumores avançados (carcinomas espinocelulares de estádio III ou IV) de cabeça e pescoço e 30 cuidadores. Foram aplicados questionários específicos (Coop/Wonca, EORTC QLQ–C30, EORTC H&N35, Coop/Wonca e *Caregiver Strain Index* – CSI) a partir de visitas de rotina ao ambulatório.

**Resultados:**

Dos 30 pacientes, 28 eram do sexo masculino e 25 apresentavam estádio IV, com idade média de 56,6 anos. 36,7% tinham o tumor primário na orofaringe e 70% sentiam dor. As escalas funcionais cognitivas, física e emocional foram as mais afetadas. Dor, fadiga e distúrbio do sono foram os sintomas mais prevalentes. Dos 30 cuidadores, 23 eram do sexo feminino e 70% eram cuidadores primários. 36,7% dos cuidadores apresentaram alto nível de estresse, principalmente relacionado à sensação de incapacidade. A comparação entre pacientes e cuidadores demonstram que os dois grupos apresentam semelhante comprometimento da qualidade de vida: aptidão física (p = 0,487), saúde mental (p = 0,615), atividades diárias (p = 0,793), atividades sociais (p = 0,301), mudança na saúde (p = 0,649), saúde geral (p = 0,168).

**Conclusão:**

O comprometimento na qualidade de vida é semelhante entre pacientes e seus cuidadores, ou seja, não só os indivíduos de fato doentes apresentam prejuízo na sua qualidade de vida, mas os seus cuidadores também e na mesma proporção.

## Introduction

Head and neck cancer accounts for 3% of all types of malignant neoplasms, and squamous cell carcinoma (SCC) represents 90% of the histological subtypes of these neoplasms. The incidence of the disease has increased in recent years, and this type of tumor is also associated with high rates of recurrence and mortality. Treatment depends on the primary tumor site and surgery plays a key role in the cure of these patients. Moreover, treatment often leads to permanent functional and esthetic sequelae in these patients.^1^, ^2^

Many studies have shown impairment related to feeding, nutrition, and pain aspects and, to a great extent, psychological problems in patients with head and neck cancer; the latter is common not only during the diagnosis and treatment but also for many subsequent years.^3^

However, little is studied regarding their caregivers’ quality of life; these are usually family members who often sacrifice their own careers or work to dedicate themselves exclusively to the patient's care. The family also has physical and emotional needs, in addition to the need for information about patient care.^4^

## Objectives

This study aims to evaluate the quality of life of patients with head and neck cancer and comparatively, of their caregivers, based on the application of validated questionnaires.

## Methods

The present study included 30 patients and their 30 respective caregivers, prospectively selected at the beginning of the study during routine consultations; no specific dates were established for study enrollment. The project was approved by the Research Ethics Committee under protocol number 447488-11, and a free and informed consent form was signed by all the study subjects.

Patients with head and neck squamous cell carcinoma stages III and IV, receiving the first treatment or with recurrence, and their informal caregivers, i.e., those who were not healthcare professionals, were included in the study. Exclusion criteria were: presence of formal caregiver (healthcare professionals); patients incapable of verbal and/or written communication, or those who refused to sign the informed consent; independent patients, i.e., those who did not require specific care from caregivers (assessed by a zero score in the “activities of daily living” (ADL) questionnaire, described below); and patient or caregiver who was unaware of the malignancy diagnosis.

The informal care is structured according to the degree of involvement of the caregiver in this task. Thus, the caregivers were further classified into three groups. Primary caregivers are primarily responsible for the patient and perform most of the tasks, such as feeding, medication, and hygiene. Secondary caregivers generally do not have the same level of responsibility as the primary caregiver, but help in some tasks, provide domestic help, and take turns with the primary caregivers. As for tertiary caregivers, although not directly involved in the care, they help other caregivers performing simple tasks, such as paying bills and doing shopping.^5^

### Used questionnaires

After identification card was filled out and the free and informed consent form was signed by both the patient and the caregiver, demographic data were collected and four questionnaires were applied: three to the selected patients (AVD, Coop/Wonca, and EORTC) and two to their caregivers (Coop/Wonca and Caregivers Strain Index–CSI). The content of these questionnaires aimed to evaluate aspects such as the overall, physical, financial, and emotional well-being.

These questionnaires were applied at once without a pre-established order in a separate room of the outpatient clinic, and the patients and their caregivers were individually surveyed. The total time of the explanation regarding the study objectives, signing of the informed consent, and questionnaire application lasted on average 30 min.

The questionnaires used for quality of life assessment were the following:

(a) Activities of Daily Living (ADL)^6^, ^7^: Used to exclude patients who were completely independent from their caregivers (score of zero shows that the patient does not require supportive care);
(b) Coop/Wonca^8^, ^9^: This questionnaire aims to produce a practical and fast tool, with a generic coverage regarding the individual's well-being and it is used to classify the patient's quality of life in the last two weeks. The individual is asked to classify his/her activities in the last two weeks. Altogether there are six items related to physical fitness, feelings, daily activities, social life, changes in health status, and overall health status. Additionally, the classification scales are illustrated with pictograms that express the following modalities, in general: excellent, very good, good, poor, and bad. The questionnaire is easily understood and well accepted by respondents. In addition, it has been widely used in general practice. Responses to the individual questionnaire items were classified from 1 to 5, where 1 is the best and 5 the worst status, and the answers were added at the end;
(c) Caregiver Strain Index (CSI)^10^: This questionnaire was specifically designed to assess the quality of life of informal caregivers. The CSI assesses 13 items considered “stressful”: inconvenience, confinement, family adjustment, change in personal plans, increased time demand, inconvenience regarding the behavior, personality change, adjustment at work, feeling of not knowing how to handle the situation, financial pressure, sleep disorders, and physical pressure. These factors are addressed as “yes-” or “no-” questions. It is believed that the CSI can be useful in preventive clinical practice or to prepare the caregivers, when using it to predict risk for a specific caregiver population. One point is attributed to each “yes” answer, up to a maximum final score of 13 points, and the higher the final score, the higher the stress level of the caregiver, and values higher than six points are equivalent to “high level of stress”;
(d) EORTC QLQ-C30 Quality of Life Index (Version 3.0)^11^: this is a quality of life questionnaire that has been internationally validated and translated into Portuguese, created by the European Organization for Research and Treatment of Cancer (EORTC), consisting of thirty questions, regarding the previous week, for which the patient must circulate numbers that represent the severity of the statements about daily activities and symptoms during that period. The H&N35 Appendix is specifically designed for patients with head and neck cancer, its symptoms, treatment, and specific quality of life. The quantification of these questionnaires followed their own guidelines and the final scores were expressed as percentages.

### Statistical analysis

Distributions were defined as non-parametric by the Kolmogorov–Smirnov test. The values obtained in the study of each quantitative variable were organized and expressed as means and standard deviations or medians and interquartile ranges (difference between percentiles 75 and 25). Absolute and relative frequencies were used for qualitative variables. The comparison between quantitative variables and two sample populations was performed by Mann–Whitney test. The statistical program SPSS version 17.0 (SPSS Inc.; Illinois, United States) was used in all analyses and the statistical significance was set at *p* < 0.05.

## Results

A total of 30 patients were interviewed, of whom 28 were males. Of the 30 respondents, 25 had stage IV disease and only four patients had recurrence. Most of them were aged 50–60 years. Most had low level of education and were married. The primary site was the oropharynx in 11 patients and the involvement of the larynx occurred in ten patients. Only five patients were undergoing chemotherapy, while 16 were using enteral nutrition and 12 had tracheostomy at the time of the interviews. Most reported feeling chronic pain and used several analgesics.

In addition to the 30 patients, their 30 corresponding caregivers were interviewed. Most caregivers were women, most of whom had a close relationship with the patient, whether spouse, child, or sibling. The caregivers’ level of education was a little better than that of patients and the monthly income was approximately 1–5 minimum wages for most of the respondents. Twenty-one individuals were considered primary caregivers. The detailed descriptive data of patients and their caregivers are shown in Table 1.

**Table 1:** Descriptive data of patients and their caregivers included in the study (*n* = 30 in each group).

Patients		Caregivers	
Variables	Results	Variables	Results
*Gender*		*Gender*	
Female	2 (6.7%)	Female	23 (76.7%)
Male	28 (93.3%)	Male	7 (23.3%)

*Age (mean)*	56.6 years	*Age (mean)*	45.4 years

*Level of schooling*		*Level of schooling*	
Illiterate	1 (3.3%)	Illiterate	1 (3.3%)
Did not finish elementary school	15 (50%)	Did not finish elementary school	6 (20%)
Finished elementary school	6 (20%)	Finished elementary school	10 (33.3%)
Did not finish high school	3 (10%)	Did not finish high school	1 (3.3%)
Finished high school	3 (10%)	Finished high school	8 (26.7%)
College/university	2 (6.6%)	College/university	4 (13.3%)

*Marital status*		*Marital status*	
Single	6 (20%)	Single	8 (26.7%)
Married	18 (60%)	Married	17 (56.7%)
Divorced	3 (10%)	Divorced	2 (6.6%)
Widowed	3 (10%)	Widowed	3 (10%)

*Other clinical characteristics*		*Kinship*	
Undergoing chemotherapy	5 (16.7%)	Partner	9 (30%)
Enteral diet	16 (53.3%)	Ex-partner	1 (3.3%)
Tracheostomy	12 (40%)	Sibling	9 (30%)
Pain	21 (70%)	Child	10 (33.3%)
		Mother	1 (3.3%)

*Primary tumor site*		*Monthly income*	
Nasopharynx	1 (3.3%)	Up to 1 minimum wage	11 (36.7%)
Oral cavity	5 (16.7%)	1–5 minimum wages	17 (56.7%)
Oropharynx	11 (36.7%)	5–10 minimum wage	2 (6.7%)
Larynx	10 (33.3%)	*Caregiver's degree*	
Hypopharynx	3 (10%)	Primary	21 (70%)
		Secondary	8 (26.7%)

The EORTC QLQ-C30 Quality of Life Index questionnaire showed that the cognitive, physical, and emotional functional scales were the most affected in the patients. Pain, fatigue, and sleep disorders (mainly insomnia) were the most prevalent symptoms. In addition, most patients reported financial difficulties during the treatment period. The EORTC QLQ-C30 data are detailed in Table 2.

**Table 2:** Basal values of EORTC–C30 questionnaire for patients.

Basal values of EORTC–C30	Median	Mean ± standard deviation^a^
*Functional scales*		
Physical function	70.00	66.00 ± 23.58
Overall function	58.33	56.67 ± 37.55
Emotional function	62.50	59.17 ± 27.01
Cognitive function	83.33	78.89 ± 19.54
Social function	50.00	59.44 ± 32.37
Overall health	58.33	54.17 ± 24.93

*Symptom scales*		
Pain	41.67	41.67 ± 36.03
Nausea and vomiting	0.00	3.33 ± 8.07
Fatigue	33.33	37.41 ± 29.25

*Isolated variables*		
Dyspnea	0.00	18.89 ± 29.92
Sleep disorder	33.33	42.22 ± 34.94
Loss of appetite	16.67	34.44 ± 41.51
Constipation	0.00	23.33 ± 31.74
Diarrhea	0.00	10.00 ± 23.41
Financial difficulties	66.67	61.11 ± 34.00

a Values in percentages.

The EORTC questionnaire H&N35 Appendix showed that most patients reported pain, ability to detect problems, difficulty in social contact, use of analgesics, and weight loss as the major factors for loss of quality of life. The EORTC H&N35 data are described in Table 3.

**Table 3:** Basal values of EORTC QLQ – H&N35 questionnaire for patients.

Variables – EORTC QLQ – H&N35	Results (%)^a^
Pain	66.11 ± 28.70
Difficulty in swallowing	50.28 ± 31.67
Ability to detect problems	68.33 ± 31.67
Speech disorders	58.15 ± 29.13
Feeding problems	50.83 ± 32.49
Problems with social contact	66.17 ± 29.97
Sexual interest	56.67 ± 40.97
Dentition	23.33 ± 34.07
Mouth opening capacity	42.22 ± 41.00
Xerostomia	38.89 ± 39.23
Thick saliva	35.56 ± 36.02
Coughing	41.11 ± 33.54
Emotional status	31.11 ± 41.00
Use of analgesics	70.00 ± 46. 61
Nutritional supplements	50.00 ± 50.85
Enteral diet	46.67 ± 50.74
Weight gain	13.33 ± 34.57
Weight loss	60.00 ± 49.83

a Mean ± standard deviation.

The Coop/Wonca questionnaire showed that most patients had poor physical fitness, moderate mental health deterioration, little difficulty in performing activities of daily living, and moderately impaired social activities. Additionally, most patients classified their overall health status as good, and little improvement was observed when the general status on the day of the interview was compared with the one two weeks before the interview. Most caregivers showed moderate physical fitness, while the impact on mental health was high. Caregivers also had little difficulty in performing activities of daily living and social activities. In general, caregivers considered their general health status as good and reported the same health status on the day of the interview and two weeks before the day of the interview.

Based on the analysis of the data obtained in the Coop/Wonca questionnaire, applied to both patients and caregivers, it was observed that there was no statistically significant difference when comparing the well-being between the two groups, that is, the quality of life impairment described above was similar between patients and their caregivers. Thus, not only the patients showed quality of life impairment but also their caregivers, and at the same proportion. These data are shown in Table 4.

**Table 4:** Comparison of the domains of the Coop/Wonca questionnaire between patients with head and neck cancer and their caregivers.

Coop/Wonca basal values	Patient^a^	Caregiver a	*p*^a^
Physical fitness	4 (3.00–4.00)	3 (2.25–4.00)	0.487
Mental health	3.5 (2.00–5.00)	4 (3.00–5.00)	0.615
Daily activities	2 (1.25–4.00)	2 (1.00–3.00)	0.793
Social activities	3 (1.00–4.00)	2 (1.00–3.00)	0.301
Changes in health	2 (2.00–3.00)	3 (2.00–3.75)	0.649
Overall health	3.5 (3.00–4.00)	3 (3.00–4.00)	0.168

a Median (interquartile range).

The CSI questionnaire also showed that 36.67% of caregivers had high levels of stress, mainly related to feelings of incapacity, changes in personal plans, and sleep disorders, as shown in Table 5.

**Table 5:** Caregiver Strain Index (CSI) questionnaire applied to caregivers.

Variables – CSI	Results
Inconvenience	10%
Confining	20%
Family adjustments	40%
Changes in personal plans	66.7%
Increase in the demand for time	50%
Inconvenience regarding the behavior	36.7%
Change in personality	46.7%
Work adjustments	46.7%
Feeling completely overwhelmed	93.3%
Financial strain	46.7%
Sleep disorders	66.7%
Physical strain	40%
Sum^a^	5.67 ± 2.89 (36.7% ≥ 7)

a Mean ± standard deviation.

## Discussion

Based on the application of the identification and quality of life questionnaires during the interviews, the study showed that the quality of life of both patients with head and neck cancer and their caregivers showed quality of life impairment, with no significant differences between the groups. This means that caregivers have a decrease in their quality of life that is proportional to the patients’, demonstrating that the disease affects not only patients but also the people around them. Currently, there are few studies that address the quality of life of caregivers of patients with head and neck cancer and there are even fewer comparative studies between the two groups.

Patients complain of pain, fatigue, and sleep disorders as major factors affecting their quality of life, while caregivers have a high level of stress related to the feeling of inability to help the patient without the adequate means to do so.

Regarding the descriptive characteristics of caregivers found in the literature, studies indicate that caregivers were predominantly female and the patient's spouse. In the present study, 76.7% of caregivers were women and only 30% were married to the patient. This fact may influence the study result, because the wives’ affective relationship make them more susceptible to emotional adjustment.^12^ However, many studies claim that the caregiver's gender is not significantly associated with psychological health.^13^, ^14^ Moreover, Ross et al.^13^ found no definitive association between the caregivers’ educational level and their quality of life, which corroborates the results of the present study, as neither the educational level, nor the social class interfered with these individuals’ quality life and emotions.

In a study on the use of quality of life questionnaires in both groups at the moment of diagnosis, it was shown that the perception of the disease was more negative in caregivers than in patients.^4^ This finding indicates that caregivers should also be evaluated periodically for the loss of their daily activities, as they have a potential risk for the development of diseases primarily related to stress. Moreover, caregivers with anxiety symptoms as a result of stress have greater difficulty in understanding the patient's needs, such as the administration of medications,^15^ which may negatively impact the patient's treatment. Similarly, Vickery et al.^16^ showed that caregivers have more psychological disorders compared to patients with head and neck cancer.

Studies suggest that a significant number (20–38%) of caregivers have emotional impairment.^13^, ^14^ Similarly, caregivers have worse mental health (e.g., high levels of depression and anxiety symptoms) than the general population^16^, ^17^ and also compared to the patients with head and neck cancer.^16^, ^18^ The literature also shows that the lifestyle changes imposed on these caregivers negatively affect their quality of life and significantly increase stress levels,^12^ a fact also observed in this study.

The present study has some limitations, as the data obtained from the questionnaires do not clarify the difference between the well-being of the two assessed study groups in the long term. Studies have shown that the period more susceptible to stress for caregivers of patients with head and neck cancer occurs during the first six months of treatment; however, there are no prospective and longitudinal studies evaluating this issue.^19^ Additionally, although the Coop/Wonca questionnaire shows changes in the same direction as the QLQ-C30 questionnaire, this tool is not sensitive enough to detect the subtle differences that the QLQ-C30 might disclose.^3^

## Conclusions

Through the analyzed data, it can be concluded that quality of life is similarly affected in both patients with head and neck cancer and their caregivers. Not only patients but also their caregivers should be evaluated for quality of life impairment and, if necessary, they should be referred for specialized care.

## Funding

Fundação de Amparo à Pesquisa do Estado de São Paulo (10.13039/501100001807FAPESP).

## Conflicts of interest

The authors declare no conflicts of interest.
